# Network meta-analysis of comparative efficacy of animal-assisted therapy vs. pet-robot therapy in the management of dementia

**DOI:** 10.3389/fnagi.2023.1095996

**Published:** 2023-05-31

**Authors:** Hongdi Du, Lin Bo, Xiaoxing Lai, Hongwei Zhu, Xiaopeng Huo

**Affiliations:** ^1^Department of Health Care, Peking Union Medical College Hospital, Chinese Academy of Medical Sciences, Beijing, China; ^2^Nursing Department, Peking Union Medical College Hospital, Chinese Academy of Medical Sciences, Beijing, China

**Keywords:** dementia, animal-assisted therapy, pet-robot therapy, agitation, network meta-analysis

## Abstract

**Objective:**

This network meta-analysis aimed to compare and rank the efficacy of animal-assisted therapy (AAT) and pet-robotic therapy (PRT) in the management of dementia.

**Methods:**

Relevant studies were identified by searching PubMed, EMBASE, the Cochrane Library, SCOPUS, and Web of Science (WoS) until October 13, 2022. Traditional meta-analysis was first conducted based on the random-effects model, then random network meta-analysis was conducted to determine the relative efficacy and rank probability of AAT and PRT.

**Results:**

Nineteen randomized controlled trials (RCTs) were included in this network meta-analysis. Network meta-analysis revealed that PRT marginally benefited agitation alleviation compared with control (standard mean difference [SMD]: −0.37, 95% confidence interval [95%CI]: −0.72 to −0.01) although both AAT and PRT did not improve cognitive function, reduce depression, and improve Quality of Life (QoL). The SUCRA probabilities indicated that PRT ranked better than AAT in agitation, cognitive function, and QoL, although there were no differences between the two therapies.

**Conclusion:**

The present network meta-analysis reveals that PRT may help alleviate agitated behaviors in people with dementia. However, future studies are warranted to establish evidence of the effectiveness of PRT and further evaluate the differences between different robot types in managing dementia.

## 1. Introduction

Dementia is a chronic degenerative encephalopathy condition characterized by cognitive and consciousness disorders, personality changes, abnormal behavior, and a decreased capacity for performing daily activities (Prince et al., 2013; Gallaway et al., 2017; Kishita et al., 2020). It is usually a subsequent condition in other diseases, such as Alzheimer's or language cortex tumors (Gale et al., 2018). According to the World Health Organization (WHO), global dementia cases will grow to 152 million by 2050 (2022). Undoubtedly, an increase in dementia cases will lead to a rise in the total cost of healthcare each year (Skaria, 2022). According to estimates, the current yearly cost of treating dementia might reach $1 trillion; by 2030, this expense will have doubled (Wimo et al., 2017).

Behavioral and psychological symptoms of dementia (BPSD), consisting of apathy, depression, agitation, aggression, anxiety, and display of behavioral deficits or excesses (Tible et al., 2017; Kang et al., 2020), are pretty prevalent in patients diagnosed with dementia (Kales et al., 2015). With the progression of their dementia, more than 90% of patients will suffer from one or more BPSD (Kales et al., 2019). Notably, BPSD has a poor impact on dementia's prognosis, referrals, hospitalization, expenditures, and quality of life (QoL), and it will also raise the burden on caregivers (Prince et al., 2013; Tible et al., 2017; Sado et al., 2018). Since there are no better therapies to slow the neurocognitive deterioration process, effective management and treatment of BPSD are crucial.

It is recommended to begin with a thorough assessment of the cause of dementia in treating cognitive degeneration (Herrmann, 2001). Non-pharmacological therapies have been recommended for the first-line treatment of BPSD in dementia in recent decades (Dyer et al., 2016). Its non-invasive nature, fewer side effects, and more affordable cost have outweighed pharmaceutical intervention (Quintavalla et al., 2021). Except for the widespread concern of overdose (Herrmann, 2001), repeated use of the drugs may also raise the risk of falls (Porsteinsson et al., 2014; Masopust et al., 2018), produce gastrointestinal problems (Tan et al., 2014; Dyer et al., 2018), and accelerate cognitive decline (Ma et al., 2014; Masopust et al., 2018).

Among numerous non-pharmacological treatments available, animal-assisted therapy (AAT) and pet-robotic therapy (PRT) have been extensively used in the management of dementia due to their significant impact on the lives of people of all ages and socioeconomic backgrounds (Hughes et al., 2020; Yu et al., 2022). Currently, several meta-analyses have evaluated the efficacy of AAT (Zafra-Tanaka et al., 2019; Batubara et al., 2022; Chen et al., 2022) and PRT (Leng et al., 2019; Lu et al., 2021; Ong et al., 2021) on BPSD in dementia, but reported conflicting results. Additionally, the difference between AAT and PRT in managing BPSD remains controversial, as only one study involving seven studies used a network meta-analysis to assess the comparative efficacy of these two therapies in reducing agitation (Leng et al., 2020). Therefore, we conducted the current network meta-analysis to comprehensively evaluate the effectiveness of AAT vs. PRT in managing BPSD in dementia by pooling direct and indirect evidence.

## 2. Materials and methods

We conducted this network meta-analysis following the guidelines from the Preferred Reporting Items for Systematic Reviews and Meta-Analyses (PRISMA) extension statement for reporting network meta-analysis (Hutton et al., 2015). Institutional review board ethical approval and patient informed consent were unnecessary for this study because all statistical analyses were completed based on the published data. The formal protocol of this network meta-analysis was not registered on any public platform.

### 2.1. Literature retrieval

Two independent authors (Hongdi Du and Lin Bo) systematically searched PubMed, EMBASE, the Cochrane Library, SCOPUS, and Web of Science (WoS) for retrieving potentially eligible studies from their inception until October 13, 2020. We developed search strategies using the MeSH terms and their synonyms, including “dementia,” “Alzheimer's disease,” “Animal-assisted therapy,” “robotics/therapy,” “robotics/therapeutic use,” and “random.” The detailed search strategies of target databases are documented in Supplementary Table 1. The search was only limited to English publications. Additionally, we manually checked references of the included studies and topic-related meta-analyses to identify those eligible studies missing from the electronic search. Any divergences between the two authors (Xiaopeng Huo and Xiaoxing Lai) were resolved by discussion until a consensus was reached.

### 2.2. Selection criteria

Based on the previous meta-analyses (Leng et al., 2020; Ong et al., 2021; Chen et al., 2022), the following inclusion criteria, which were developed based on the PICOS acronym, were used for guiding the selection of potentially eligible studies: (a) adult patients were diagnosed with dementia, (b) patients in the experimental group received AAT or PRT, (c) patients in the control group received usual care or one of AAT and PRT, (d) studies reported at least one of agitation, cognitive function, depression, and the QoL, and (e) randomized controlled trials published with full texts.

Studies were excluded if they met the following criteria: (a) repeated reports of the same study; (b) studies assessing the robot's acceptability to participants, (c) studies were conducted to evaluate companion robot's development and usability, (d) lack of data to perform the network meta-analysis; and (e) studies using ineligible designs such as review articles, conference abstracts, qualitative studies, contemplated articles, or animal studies.

### 2.3. Study selection

We used EndNote X9.2 (Clarivate Analytics) to manage records retrieved from electronic retrieval. EndNote software was first used to exclude duplicates before evaluating the eligibility of these records. Two independent authors (Hongdi Du and Xiaoxing Lai) then evaluated the eligibility of all unique records according to the eligibility criteria by preliminarily screening their titles and abstracts. For those studies retained after screening the titles and abstracts, their eligibility was evaluated by screening full texts. Any divergences between the two authors (Xiaopeng Huo and Hongwei Zhu) were resolved by discussion until a consensus was reached.

### 2.4. Data extraction

Two independent authors (Lin Bo and Hongwei Zhu) used a pre-designed data extraction sheet to complete data extraction. Specifically, the following information was extracted from the included studies: the first author's surname, country, study design, setting, sample size, participants' mean age, dementia stage, details of interventions, outcomes of interest, and detailed information of quality assessment. We extracted the data of the final follow-up for meta-analysis. We used the recognized formula to estimate mean and standard deviation according to median and range, standard error, or interquartile range (Wan et al., 2014). Any divergences between the two authors (Hongdi Du and Xiaopeng Huo) were resolved by discussion until a consensus was reached.

### 2.5. Outcomes of interest

We considered agitation the primary outcome in this network meta-analysis, while cognitive function, depression, and QoL were secondary outcomes. The instruments used to measure the outcomes were not limited but must have acceptable reliability and validity.

### 2.6. Evidence structure

The evidence structures of all outcomes were constructed using a network plot. The network plot had two essential elements: a node and a line. The node represented the intervention and was weighted using the accumulated sample size; however, the line represented the comparison that directly compared two interventions, and the width of the line was weighted by using the number of eligible studies (Salanti et al., 2011).

### 2.7. Risk of bias assessment

Two authors (Hongdi Du and Lin Bo) independently assessed the methodological quality of the included studies using the Cochrane risk of bias assessment tool (Higgins et al., 2011). Specifically, the methodological quality of each study was determined by evaluating the following seven items: random sequence generation, allocation concealment, blinding of participants and personnel, blinding of outcome assessor, incomplete outcome data, selective reporting, and other bias. For each item, one of three labels would be assigned, including “low,” “high,” or “unclear” risk. The overall level of methodological quality of each study might be rated as “high” level if all items were labeled with “low” risk, as “low” level if at least one of the seven items were labeled with “high” risk, or “moderate” level if at least one the seven items were labeled with “unclear” risk but no item was labeled with “high” risk. Any divergences between the two authors (Xiaopeng Huo and Xiaoxing Lai) were resolved by discussion until a consensus was reached.

### 2.8. Statistical analysis

#### 2.8.1. Traditional meta-analysis

Standard mean difference (SMD) with the corresponding 95% confidence interval (CI) was used to express the pooled estimates because all outcomes were continuous variables in this study but were measured using various tools. We first conducted a traditional meta-analysis to evaluate the efficacy of AAT or PRT in the management of participants with dementia (DerSimonian and Laird, 1986). Statistical heterogeneity between the included studies was assessed using the Cochrane *Q* statistic (Bowden et al., 2011) and Higgins' inconsistency factor (*I*^2^) (Higgins and Thompson, 2002). Statistical heterogeneity was considered significant if *P* < 0.1 and *I*^2^ > 50%. Nevertheless, meta-analysis was conducted based on the random-effects model because it was not rational to deny the variations between studies (Biggerstaff and Tweedie, 1997).

#### 2.8.2. Network meta-analysis

Then, a network meta-analysis based on a random-effects model was conducted to determine the difference between AAT and PRT in terms of all outcomes of interest (Lu and Ades, 2004; Dias and Caldwell, 2019). First, the transitivity examination was first conducted to determine whether it is rational to conduct a network meta-analysis based on the major factors (Salanti, 2012; Cipriani et al., 2013), including publication year, origin, sample size, mean age, gender ratio, and dementia stage. Then, global inconsistency was examined using the design-by-treatment interaction method (Tu, 2015), and local inconsistency was tested using the node-splitting method when both direct and indirect evidence were available (Higgins et al., 2012). Third, we used the node-splitting method to assess the closed-loop inconsistency (Lu and Ades, 2006; Yu-Kang, 2016). Fourth, we calculated the surface under the cumulative ranking curve (SUCRA) probabilities to rank AAT and PRT, and a higher SUCRA value indicated a better ranking (Mbuagbaw et al., 2017). Finally, a comparison-adjusted funnel plot was used to assess the small-sample effect (Sterne et al., 2001). Additionally, Egger's and Begg's tests were used to evaluate publication bias quantitatively (Palma Perez and Delgado Rodriguez, 2006; Page et al., 2018). We conducted all analyses using STATA 14.0 (StataCorp LP, College Station, Texas, USA) (Chaimani et al., 2013; White, 2017).

## 3. Results

### 3.1. Selection of eligible studies

**Figure 1:**
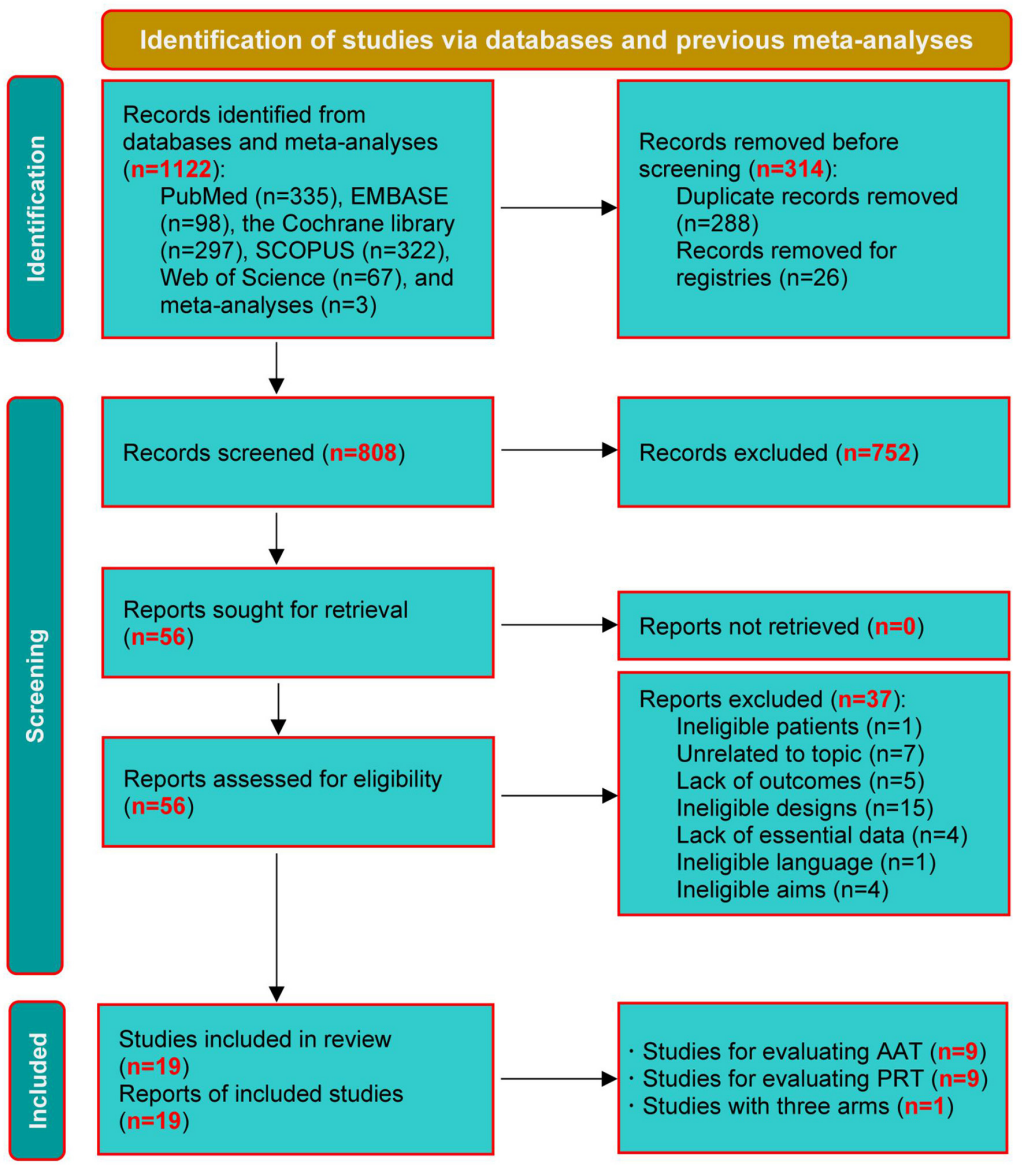
PRISMA diagram of study retrieval and selection. AAT, animal-assisted therapy; PRT, per-robot therapy.

A total of 1,122 relevant studies were retrieved from the databases and the published meta-analyses. Before conducting the eligibility evaluation, 288 duplicate studies and 26 registries were excluded. After excluding 752 ineligible studies based on title and abstract screening, 56 were screened by checking the full texts. Finally, 19 studies (Moyle et al., 2013, 2017, 2019; Robinson et al., 2013; Travers et al., 2013; Bono et al., 2015; Friedmann et al., 2015; Jøranson et al., 2015, 2016; Valenti Soler et al., 2015; Pope et al., 2016; Olsen et al., 2016a,b; Liang et al., 2017; Petersen et al., 2017; Pu et al., 2020; Briones et al., 2021; Quintavalla et al., 2021; Vegue Parra et al., 2021) were included in this network meta-analysis after excluding 37 ineligible studies due to ineligible patients (*n* = 1), unrelated to the topic (*n* = 7), lack of outcomes (*n* = 5), ineligible study designs (*n* = 15), lack of essential data (*n* = 4), ineligible language (*n* = 1), and ineligible aims (*n* = 4). The process of study selection is depicted in Figure 1.

### 3.2. Basic characteristics of the included studies

We summarized the basic characteristics of the included studies in Table 1, and listed the detailed information on AAT and PRT of the included studies in Supplementary Table 2. The outcomes and measurements of the included studies are documented in Supplementary Table 3. Overall, 18 studies (Moyle et al., 2013, 2017, 2019; Robinson et al., 2013; Travers et al., 2013; Bono et al., 2015; Friedmann et al., 2015; Jøranson et al., 2015, 2016; Pope et al., 2016; Olsen et al., 2016a,b; Liang et al., 2017; Petersen et al., 2017; Pu et al., 2020; Briones et al., 2021; Quintavalla et al., 2021; Vegue Parra et al., 2021) were two-arm design and one study (Valenti Soler et al., 2015) was three-arm design, and all studies were published between 2013 and 2021. The sample size of the included studies ranged from 24 to 334, accumulating a total sample size of 1464. Nine studies (Moyle et al., 2013, 2019; Friedmann et al., 2015; Jøranson et al., 2015; Pope et al., 2016; Olsen et al., 2016b; Liang et al., 2017; Pu et al., 2020) reported the data of agitation. Five studies (Bono et al., 2015; Jøranson et al., 2016; Liang et al., 2017; Quintavalla et al., 2021; Vegue Parra et al., 2021) reported the data on cognitive function, and eleven studies (Moyle et al., 2013; Robinson et al., 2013; Travers et al., 2013; Bono et al., 2015; Friedmann et al., 2015; Jøranson et al., 2015; Olsen et al., 2016b; Liang et al., 2017; Petersen et al., 2017; Pu et al., 2020; Vegue Parra et al., 2021) reported the data of depression. Eight studies (Moyle et al., 2013; Robinson et al., 2013; Travers et al., 2013; Valenti Soler et al., 2015; Jøranson et al., 2016; Olsen et al., 2016a,b; Briones et al., 2021) reported the data of QoL. The evidence structures of all outcomes are depicted in Supplementary Figure 1.

**Table 1:** Basic characteristics of the included studies.

**Study**	**Country**	**Setting**	**Group**	**Participants at baseline, *n***	**Participants for analysis, *n***	**Genderratio (M/F), *n***	**Mean age, years**	**Tool for dementia assessment**	**Dementia stage**
Travers et al. (2013)	Australia	Long-term care facilities	Dog	34	27	8/19	84.9 ± 6.1	MSE-3MS (0–100)	58.1 ± 19.8
			Control	33	28	4/24	85.1 ± 6.6		59.8 ± 17.2
Bono et al. (2015)	Italy	Long-term care unit	Dog	16	12	n.a.	82.1 ± 6.2	MMSE (0–30)	17.3 ± 3.7
			Control	16	12	n.a.	78.3 ± 10.3		17.3 ± 3.7
Friedmann et al. (2015)	USA	Nursing home	Dog	22	19	7/15	79.6 ± 9.7	MMSE (0–30)	14.6 ± 5.2
			Control	18	18	4/14	82.1 ± 8.4		13.9 ± 3.5
Olsen et al. (2016a)	Norway	Nursing home	Dog	42	41	18/23	84	MMSE (0–30)	≤25
			Control	38	38	14/24	81.7		
Olsen et al. (2016b)	Norway	Day care center	Dog	30	25	9/17	82.9 ± 8.5	MMSE (0–30)	13.8 ± 6.6
			Control	28	26	11/15	84.1 ± 6.7		13.8 ± 6.6
Briones et al. (2021)	Spain	Public care home	Dog	16	16	3/13	89.3 ± 1.9	MMSE-S (0–30)	16.7 ± 1.1
			Control	18	18	6/12	88.2 ± 1.1		16.7 ± 1.2
Pope et al. (2016)	USA	Long-term care facilities	Dog	44	44	24/20	79.8	n.a.	n.a.
			Control	44	44	24/20			
Vegue Parra et al. (2021)	Spain	Senior centers	Dog	186	171	n.a.	>65	MMSE (0–30)	<25
			Control	185	163	n.a.	>65		
Quintavalla et al. (2021)	Italy	Day-care center	Dog	30	30	8/22	>60	MMSE (0–30)	14.35 ± 7.16
			Control	10	10	3/7	>60		21.94 ± 6.39
Jøranson et al. (2015)	Norway	Nursing home	PARO	30	27	8/19	83.9 ± 7.2	MMSE (0–30)	≤25
			Control	30	26	10/16	84.1 ± 6.7		
Jøranson et al. (2016)	Norway	Nursing home	PARO	53	27	8/19	83.9 ± 7.2	MMSE (0–30)	≤25
			Control	53	26	10/16	84.1 ± 6.7		
Liang et al. (2017)	New Zealand	Day care center	PARO	15	13	n.a.	67-98	n.a.	n.a.
			Control	15	11	n.a.			
Moyle et al. (2013)	Australia	Long-term care facilities	PARO	18	18	n.a.	85.3 ± 8.4	n.a.	n.a.
			Control	18	18	n.a.			
Moyle et al. (2017)	Australia	Long-term care facilities	PARO	138	138	37/101	84.0 ± 8.4	RUDAS (0–30)	6.5 ± 6.5
			Control	137	137	38/99	85.0 ± 7.1		8.3 ± 7.2
Moyle et al. (2019)	Australia	Long-term care facilities	Lifelike doll	18	18	0/18	86.1 ± 8.6	MMSE (0–30)	4.9 ± 4.8
			Control	17	15	0/15	89.7 ± 8.4		5.8 ± 4.9
Petersen et al. (2017)	USA	Dementia unit	PARO	35	35	8/27	83.5 ± 5.8	GDS (1–7)	5.6 ± 0.8
			Control	26	26	6/20	83.3 ± 6.0		5.3 ± 1.0
Pu et al. (2020)	Australia	Long-term care facilities	PARO	21	21	3/18	86.5 ± 8.8	MMSE (0–30)	7.7 ± 7.8
			Control	22	22	10/12	85.6 ± 5.8		12.1 ± 7.8
Robinson et al. (2013)	New Zealand	Hospital & rest home	PARO	20	17	n.a.	55-100	n.a.	n.a.
			Control	20	17	n.a.			
Valenti Soler et al. (2015)	Spain	Nursing home	Dog	36	36	n.a.	84.7	n.a.	n.a.
			PARO	42	42	n.a.		GDS (1–7)	3.2 ± 5.0
			Control	32	32	n.a.			3.6 ± 5.4

MSE-3MS, Modified Mini-Mental State Exam; MMSE, Mini Mental State Examination; RUDAS, The Rowland Universal Dementia Assessment Scale; GDS, Global Deterioration Scale. n.a., not available.

### 3.3. Risk of bias of eligible studies

All included studies correctly generated random sequences, but only five (Jøranson et al., 2015, 2016; Liang et al., 2017; Moyle et al., 2017, 2019) adequately reported the information on allocation concealment. A total of16 studies (Moyle et al., 2013; Travers et al., 2013; Bono et al., 2015; Friedmann et al., 2015; Jøranson et al., 2015, 2016; Valenti Soler et al., 2015; Pope et al., 2016; Olsen et al., 2016a,b; Liang et al., 2017; Petersen et al., 2017; Pu et al., 2020; Briones et al., 2021; Quintavalla et al., 2021; Vegue Parra et al., 2021) did not blind participants and personnel, and 12 studies (Robinson et al., 2013; Friedmann et al., 2015; Jøranson et al., 2015, 2016; Pope et al., 2016; Olsen et al., 2016a,b; Liang et al., 2017; Moyle et al., 2017, 2019; Petersen et al., 2017; Pu et al., 2020) did not blind outcome assessors. Six studies (Moyle et al., 2013; Robinson et al., 2013; Travers et al., 2013; Bono et al., 2015; Olsen et al., 2016a) had incomplete data but did not use an appropriate approach to conduct data analysis. Five studies (Moyle et al., 2013; Travers et al., 2013; Jøranson et al., 2015, 2016; Valenti Soler et al., 2015) were labeled with high risk due to selective outcome reporting. Four studies (Pope et al., 2016; Olsen et al., 2016b; Moyle et al., 2019; Briones et al., 2021) had high risk in other bias sources. The risk-of-bias assessment of the included studies is displayed in Supplementary Figure 2. Overall, the methodological quality of the included studies was low.

### 3.4. Transitivity assessment

Transitivity between the included studies was assessed based on six major factors: origin, publication year, sample size, the mean age of participants, gender ratio, and dementia stage. As shown in Supplementary Table 4, the distributions of these six factors were not significantly different in all comparisons.

### 3.5. Inconsistency examination

As shown in Supplementary Figure 3, the results indicated non-significant global consistency for cognitive function (*P* = 0.441) and depression (*P* = 0.608) but significant global inconsistency for agitation (*P* = 0.045). Nevertheless, we selected an inconsistency model for network meta-analysis of these three outcomes because no direct evidence was available for cognitive function, agitation, and depression. For QoL, the global inconsistency examination was not statistically significant (*P* = 0.940). Meanwhile, the local inconsistency examination for QoL was insignificant, reporting a P of 0.749 for comparing AAT with control, a P of 0.730 for comparing PRT with control, and a *P* of 0.686 for the comparison of AAT with PRT. Therefore, the consistency model was used for the network meta-analysis of QoL.

### 3.6. Meta-analysis of agitation

**Figure 2:**
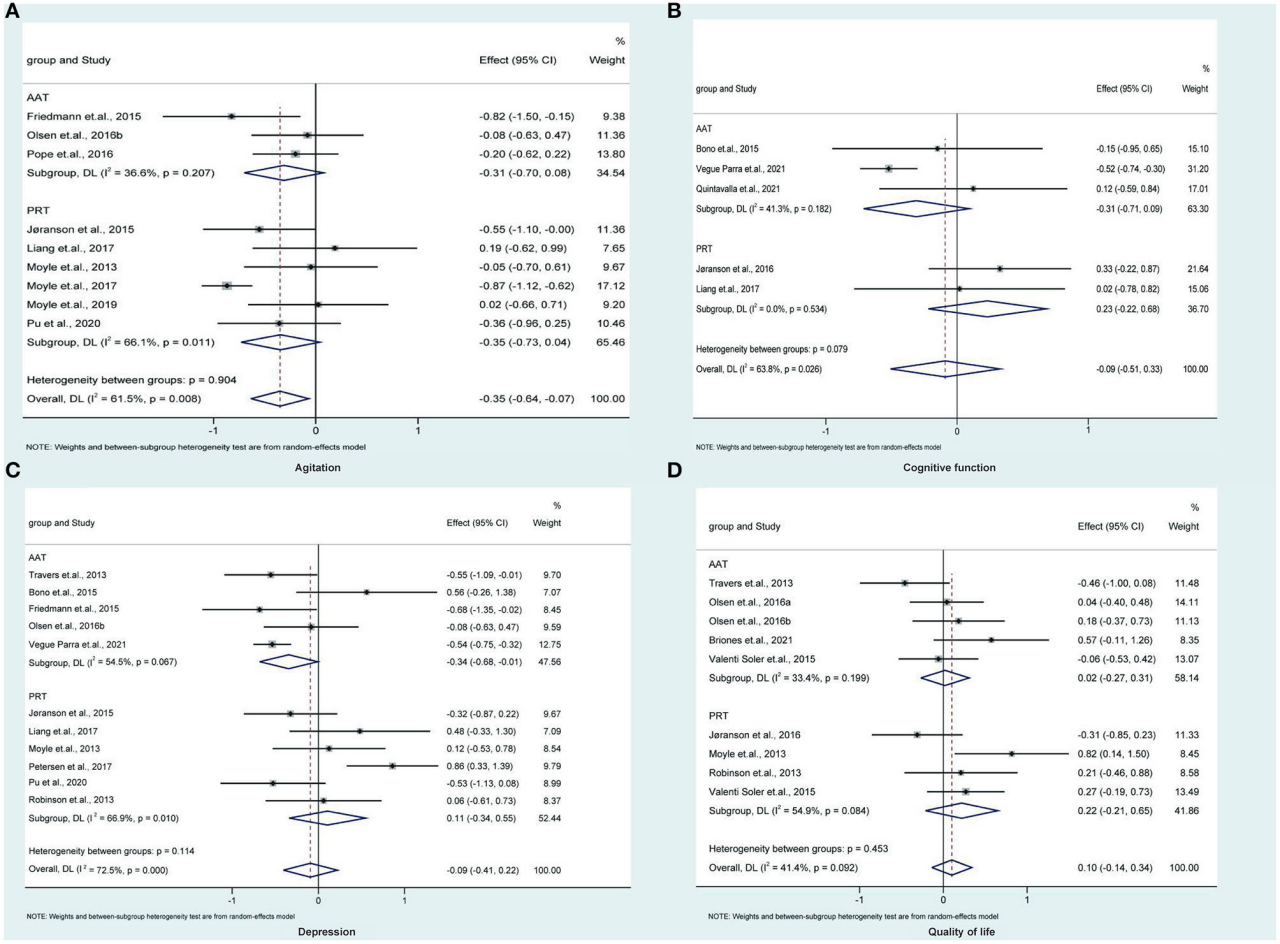
Traditional direct meta-analysis of AAT and PRT in terms of agitation **(A)**, cognitive function **(B)**, depression **(C)**, and quality of life **(D)**. AAT, animal-assisted therapy; PRT, per-robot therapy; CI, confidence interval; DL, Dersimonian-Laird.

**Figure 3:**
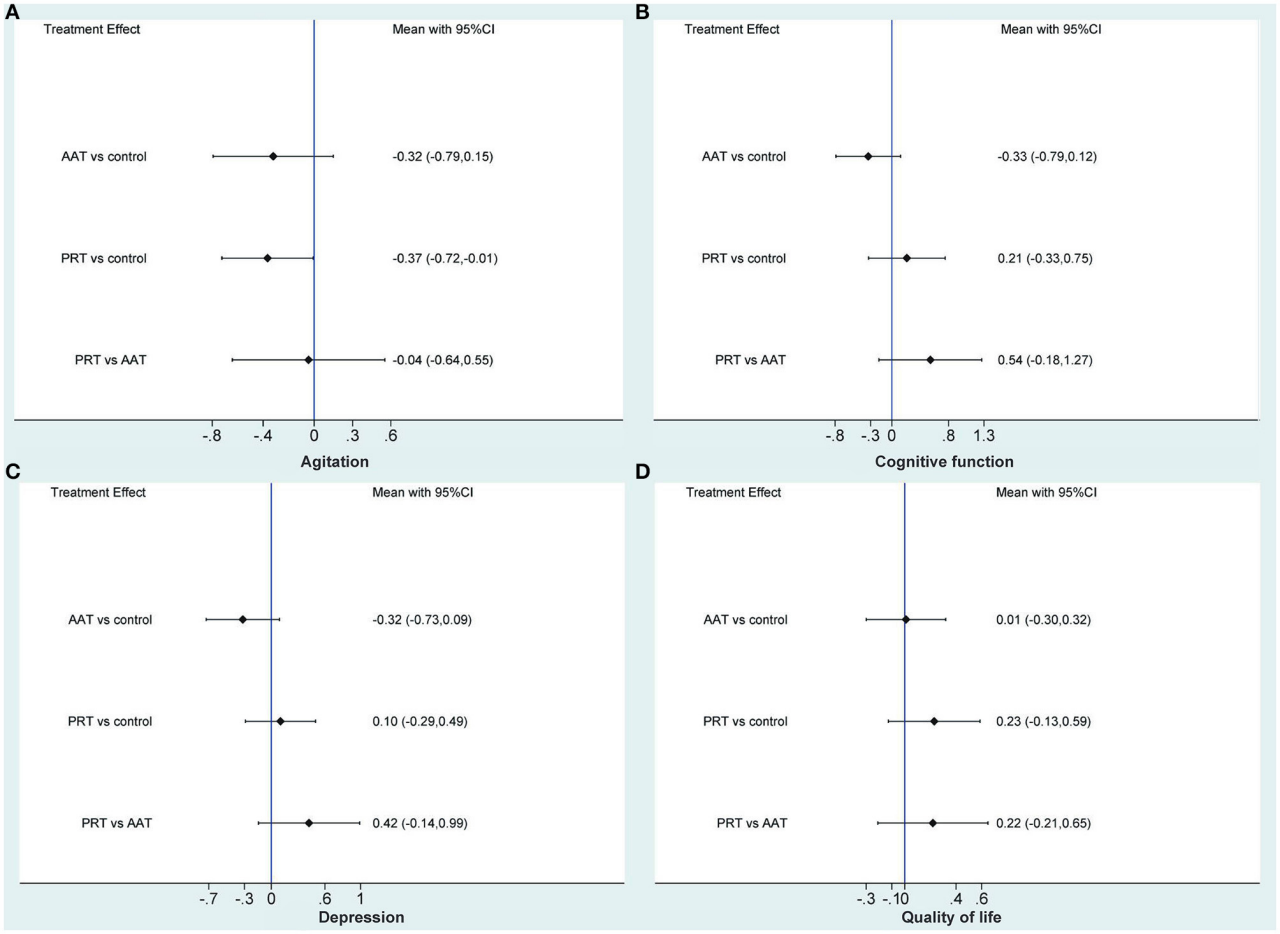
Network meta-analysis of AAT and PRT in terms of agitation **(A)**, cognitive function **(B)**, depression **(C)**, and quality of life **(D)**. AAT, animal-assisted therapy; PRT, per-robot therapy; CI, confidence interval.

Three (Friedmann et al., 2015; Pope et al., 2016; Olsen et al., 2016b) and six (Moyle et al., 2013, 2017, 2019; Jøranson et al., 2015; Liang et al., 2017; Pu et al., 2020) studies compared AAT and PRT with control, respectively. Traditional meta-analysis revealed that, as shown in Figure 2A, AAT (SMD: −0.31, 95%CI: −0.70 to 0.08, *P*=0.114) and PRT (SMD: −0.35, 95%CI: −0.73 to 0.04, *P* = 0.078) did not significantly alleviate agitated behaviors of participants of dementia. As shown in Figure 3A, the network meta-analysis results revealed that AAT did not considerably alleviate agitation (SMD: −0.32; 95% CI: −0.79 to 0.15), but PRT marginally benefited agitation alleviation (SMD: −0.37; 95% CI: −0.72 to −0.01). Nevertheless, network meta-analysis revealed that AAT and PRT did not differ significantly in agitation (SMD: −0.04, 95% CI: −0.64 to 0.55).

### 3.7. Meta-analysis of cognitive function

Three (Bono et al., 2015; Quintavalla et al., 2021; Vegue Parra et al., 2021) and two (Jøranson et al., 2016; Liang et al., 2017) studies compared AAT and PRT with control, respectively. Traditional meta-analysis revealed that, as shown in Figure 2B, AAT (SMD: −0.31, 95%CI: −0.71 to 0.09, *P* = 0.130) and PRT (SMD: 0.23, 95%CI: −0.22 to 0.68, *P* = 0.316) did not significantly improve cognitive function of participants of dementia. As shown in Figure 3B, the results of the traditional meta-analysis were further confirmed by network meta-analysis. Furthermore, network meta-analysis revealed that AAT and PRT did not differ significantly in cognitive function (SMD: 0.54, 95% CI: −0.18 to 1.27).

### 3.8. Meta-analysis of depression

Five (Travers et al., 2013; Bono et al., 2015; Friedmann et al., 2015; Olsen et al., 2016b; Vegue Parra et al., 2021) and six (Moyle et al., 2013; Robinson et al., 2013; Jøranson et al., 2015; Liang et al., 2017; Petersen et al., 2017; Pu et al., 2020) studies compared AAT and PRT with control, respectively. Traditional meta-analysis revealed that, as shown in Figure 2C, AAT (SMD: −0.34, 95%CI: −0.68 to −0.01, *P* = 0.045) rather than PRT (SMD: 0.11, 95%CI: −0.34 to 0.55, *P* = 0.641) marginally benefited depression reduction. As shown in Figure 3C, however, network meta-analysis revealed that both AAT (SMD: −0.32; 95% CI: −0.73 to 0.09) and PRT (SMD: 0.10; 95% CI: −0.29 to 0.49) did not significantly reduce depression. Furthermore, network meta-analysis also revealed that AAT and PRT did not differ significantly in terms of agitation (SMD: 0.42, 95% CI: −0.14 to 0.99).

### 3.9. Meta-analysis of QoL

Five (Travers et al., 2013; Valenti Soler et al., 2015; Olsen et al., 2016a,b; Briones et al., 2021) and four (Moyle et al., 2013; Robinson et al., 2013; Valenti Soler et al., 2015; Jøranson et al., 2016) studies compared AAT and PRT with control, respectively. Traditional meta-analysis revealed that, as shown in Figure 2D, both AAT (SMD: 0.02, 95% CI: −0.27 to 0.31, *P* = 0.894) and PRT (SMD: 0.22, 95%CI:−0.21 to 0.65, *P* = 0.322) did not significantly improve QoL. As shown in Figure 3D, the results of the traditional meta-analysis were further confirmed by network meta-analysis. Furthermore, network meta-analysis revealed that AAT and PRT did not differ significantly in QoL (SMD: 0.22, 95% CI: −0.21 to 0.65).

### 3.10. Ranking probability

As shown in Supplementary Figure 4, the SUCRA probability of PRT was 77.7% in agitation, 85.4% in cognitive function, 18.4% in depression, and 87.1% in QoL; however, the SUCRA probability of AAT was 66.7% in agitation, 7.4% in cognitive function, 93.4% in depression, and 34.3% in QoL. Overall, the PRT was probably a better choice than the AAT because it ranks better in most outcomes, but more studies should validate it.

### 3.11. Heterogeneity assessment

For AAT, the local statistical heterogeneity for meta-analysis of agitation, cognitive function, depression, and QoL was 36.6, 41.3, 54.5, and 33.4%, respectively. However, for PRT, the local statistical heterogeneity for meta-analysis of agitation, cognitive function, depression, and QoL was 66.1, 0.0, 66.9, and 54.9%, respectively. The global statistical heterogeneity was 61.5, 63.8, 72.5, and 41.4% for agitation, cognitive function, depression, and QoL, respectively.

### 3.12. Publication bias

As shown in Supplementary Figure 5, the assumption of publication bias was not detected for all outcomes because the outlines of all funnel plots were symmetric. However, Begg's and Egger's tests revealed publication bias for agitation (*P* = 0.013 for Beeg's test and *P* < 0.000 for Egger's test) and cognitive function (*P* = 1.000 for Beeg's test and *P* = 0.008 for Egger's test) although revealed no publication bias for depression (*P* = 0.297 for Beeg's test and *P* = 0.225 for Egger's test), and QoL (*P* = 0.233 for Beeg's test and *P* = 0.270 for Egger's test).

## 4. Discussion

Dementia has emerged as a major global public health problem and will continue to be a public health burden in the future (Frankish and Horton, 2017). Therefore, it is crucial to improve the management and treatment of BPSD. AAT and PRT have been widely used to manage dementia; however, the difference in efficacy between the two therapies in managing BPSD remains controversial. In this network meta-analysis, 19 eligible RCTs were included to estimate the relative efficacy of AAT vs. PRT. The results revealed that compared with control, PRTsignificantly alleviated agitation in dementia, although AAT and PRT did not improve cognitive function, reduce depression, or improve QoL. The SUCRA probabilities indicated that PRT ranked better than AAT in terms of agitation, cognitive function, and QoL, although the two therapies did not differ significantly in these outcomes.

To date, several meta-analyses (Leng et al., 2019, 2020; Zafra-Tanaka et al., 2019; Park et al., 2020; Lu et al., 2021; Ong et al., 2021; Batubara et al., 2022; Chen et al., 2022) have evaluated the efficacy of AAT and PRT on BPSD in dementia. Of these meta-analyses, three (Zafra-Tanaka et al., 2019; Batubara et al., 2022; Chen et al., 2022) focused on the role of AAT, three (Leng et al., 2019; Lu et al., 2021; Ong et al., 2021) focused on the role of PRT, and one (Park et al., 2020) simultaneously focused on roles of AAT and PRT. Three meta-analyses (Zafra-Tanaka et al., 2019; Batubara et al., 2022; Chen et al., 2022) focusing on AAT revealed that AAT had no effect on cognitive function, agitation and QoL but had a positive effect on depression in patients with dementia. It is important to note that these meta-analyses did not include all eligible studies, and some even simultaneously pooled the results of RCTs and non-RCTs. Therefore, the results of these meta-analyses should be interpreted with caution. In this study, the traditional meta-analysis revealed a positive effect of AAT on depression; however, network meta-analysis significantly changed the results of the traditional meta-analysis. So, it is speculated that insufficient statistical power might be the major contributor to the false positive result. Another three meta-analyses focusing on PRT (Leng et al., 2019; Lu et al., 2021; Ong et al., 2021) consistently revealed that PRT significantly alleviated agitation in dementia. Additionally, of these three meta-analyses, two (Lu et al., 2021; Ong et al., 2021) showed no positive effect of PRT on depression and QoL, but one (Leng et al., 2019) showed a positive effect of PRT on depression. Similarly, some eligible studies have been missed from these meta-analyses, thus significantly decreasing the robustness and reliability of their findings.

To determine the relative efficacy of different non-pharmacological interventions in managing agitation in dementia, Leng et al. did a Bayesian network meta-analysis in 2020 (Leng et al., 2020). Their network meta-analysis included four studies focusing on AAT and three studies focusing on PRT to calculate the relative efficacy of AAT versus PRT. The pooled result showed that AAT was not significantly different from PRT in reducing agitation (SMD: 0.41, 95%CI: −6.32 to 7.40), which was consistent with the results of this network meta-analysis. However, more outcomes were assessed in the present network meta-analysis than in previous network meta-analyses, including cognitive function, depression, and QoL, thus inevitably increasing the reference value of our network meta-analysis. In addition, it cannot be ignored that more eligible studies were included in this network meta-analysis, thus significantly increasing statistical power. Therefore, our findings should be prioritized for decision-making.

The current network meta-analysis yields some promising findings because it has unique strengths. First, direct and indirect evidence were pooled simultaneously to increase the statistical power significantly. Seconds, all eligible studies included in this network meta-analysis were RCTs, which benefited from decreasing the negative impact of confounding factors on the pooled results. Third, traditional and network meta-analyses were simultaneously conducted to estimate the efficacy of AAT and PRT, which benefited in determining whether the results from the traditional meta-analysis were false positive or negative, resulting from insufficient statistical power. Finally, we calculated SUCRA probabilities for AAT and PRT in all outcomes to detect subtle differences between these two therapies, which benefited in determining which might be the preferred strategy for a particular outcome.

This network meta-analysis has some limitations. First, Egger's and Begg's tests confirmed publication bias for meta-analyses of cognitive function and agitation, so it is impossible to deny that a small sample effect may negatively affect the reliability of the pooled results. Seconds, although 19 eligible studies were included in this network meta-analysis, it is unavoidable that insufficient sample size may compromise the robustness of our findings. Third, although the 19 eligible studies included in this network meta-analysis were RCTs, the overall methodological quality of these included RCTs was low according to the risk of bias assessment. Therefore, poor methodological quality will inevitably challenge the reliability of our findings. Fourth, details of AAT or PRT varied among the included studies. Although the efficacy of these two therapies was closely related to the frequency and duration of treatment, the limited number of included studies made it inappropriate to divide the two therapies into groups based on frequency and duration of treatment. Smaller groups meant limited data, which would seriously compromise the accuracy and reliability of results. Fifth, we estimated the relative efficacy of AAT and PRT in most outcomes based only on indirect evidence, which inevitably compromised the reliability of pooled results. Therefore, more studies directly comparing AAT and PRT are needed to determine how these two therapies differ in managing dementia. Finally, the cultural and architectural environment may also affect the effects of AAT and PRT on dementia. However, all eligible studies did not provide this information; therefore, it is necessary to evaluate the effects of the information on the effectiveness of AAT and PRT in managing dementia in future studies.

## 5. Conclusion

We conducted an exhaustive comparison of the efficacy of AAT and PRT on the common BPSD in dementia. Network meta-analysis shows that PRT alleviates agitation in patients diagnosed with dementia compared to control, although neither AAT nor PRT does not improve cognitive function, reduces depression, and improves QoL. The SUCRA probabilities indicate that PRT ranks better than AAT regarding agitation, cognitive function, and QoL, although the two therapies do not differ significantly in these outcomes. Therefore, in clinical practice, PRT may be a potential strategy for managing agitated behaviors in dementia to reduce the burden on healthcare systems and caregivers. In addition, more studies directly comparing AAT with PRT are needed to establish evidence of effectiveness, and further studies are also warranted to determine the difference between different robot types in the management of dementia.

## Data availability statement

The original contributions presented in the study are included in the article/Supplementary material, further inquiries can be directed to the corresponding authors.

## Author contributions

HD and LB carried out the studies, participated in collecting data, and drafted the manuscript. XL and HZ performed the statistical analysis and participated in its design. HD and XH participated in acquiring, analyzing, interpreting data, and drafting the manuscript. All authors read and approved the final manuscript.
