# Supplementary material for: Network meta-analysis of comparative efficacy of animal-assisted therapy vs. pet-robot therapy in the management of dementia
Source: Front Aging Neurosci. 2023 May 31;15:1095996. doi: 10.3389/fnagi.2023.1095996 (PMC10264590; doi:10.3389/fnagi.2023.1095996)
Supplement: Supplementary file 1 [file Table_1.DOCX]

**Supplementary Table 1.** Detailed search strategies of target databases.

*PubMed*

| No. | Search Details | Results |
| --- | --- | --- |
| 14 | ("Dementia"[MeSH Terms] OR "Alzheimer Disease"[MeSH Terms] OR ("Dementia"[Title/Abstract] OR "Amentia"[Title/Abstract] OR "Alzheimer Disease"[Title/Abstract] OR "alzheimer s disease"[Title/Abstract] OR "alzheimer sclerosis"[Title/Abstract] OR "alzheimer syndrome"[Title/Abstract])) AND ("Animal Assisted Therapy"[MeSH Terms] OR "Pets"[MeSH Terms] OR ("animal assisted therapies"[Title/Abstract] OR "animal facilitated therapy"[Title/Abstract] OR (("animals"[MeSH Terms:noexp] OR "Animal"[All Fields]) AND "facilitated therapies"[Title/Abstract]) OR "Animal Assisted Therapy"[Title/Abstract] OR "pet therapy"[Title/Abstract] OR "pet therapies"[Title/Abstract] OR "pet facilitated therapy"[Title/Abstract] OR ("Pet"[All Fields] AND "facilitated therapies"[Title/Abstract]) OR "pet assisted therapy"[Title/Abstract] OR ("Pet-Assisted"[All Fields] AND "Therapies"[Title/Abstract]) OR "pet assisted therapy"[Title/Abstract] OR ("Pet"[All Fields] AND "assisted therapies"[Title/Abstract]) OR "Pet"[Title/Abstract] OR "companion animal"[Title/Abstract] OR "companion cat"[Title/Abstract] OR "companion dog"[Title/Abstract]) OR ("robotics/therapeutic use"[MeSH Terms] OR "robotics/therapy"[MeSH Terms] OR ("robot therapy"[Title/Abstract] OR "robot therapy sessions"[Title/Abstract] OR "social robot"[Title/Abstract] OR "socially assistive robot"[Title/Abstract] OR "robotic pet"[Title/Abstract] OR "robotic therapy"[Title/Abstract] OR "robot assisted therapy"[Title/Abstract] OR "robot assisted therapies"[Title/Abstract]))) AND ("Randomized Controlled Trial"[Publication Type] OR "Randomized Controlled Trials as Topic"[MeSH Terms] OR "Random Allocation"[MeSH Terms] OR "random*"[All Fields]) | 335 |
| 13 | "Randomized Controlled Trial"[Publication Type] OR "Randomized Controlled Trials as Topic"[MeSH Terms] OR "Random Allocation"[MeSH Terms] OR "random*"[All Fields] | 1,599,873 |
| 12 | "random*"[All Fields] | 1,598,371 |
| 11 | "Randomized Controlled Trial"[Publication Type] OR "Randomized Controlled Trials as Topic"[MeSH Terms] OR "Random Allocation"[MeSH Terms] | 819,990 |
| 10 | "Animal Assisted Therapy"[MeSH Terms] OR "Pets"[MeSH Terms] OR ("animal assisted therapies"[Title/Abstract] OR "animal facilitated therapy"[Title/Abstract] OR (("animals"[MeSH Terms:noexp] OR "Animal"[All Fields]) AND "facilitated therapies"[Title/Abstract]) OR "Animal Assisted Therapy"[Title/Abstract] OR "pet therapy"[Title/Abstract] OR "pet therapies"[Title/Abstract] OR "pet facilitated therapy"[Title/Abstract] OR ("Pet"[All Fields] AND "facilitated therapies"[Title/Abstract]) OR "pet assisted therapy"[Title/Abstract] OR ("Pet-Assisted"[All Fields] AND "Therapies"[Title/Abstract]) OR "pet assisted therapy"[Title/Abstract] OR ("Pet"[All Fields] AND "assisted therapies"[Title/Abstract]) OR "Pet"[Title/Abstract] OR "companion animal"[Title/Abstract] OR "companion cat"[Title/Abstract] OR "companion dog"[Title/Abstract]) OR ("robotics/therapeutic use"[MeSH Terms] OR "robotics/therapy"[MeSH Terms] OR ("robot therapy"[Title/Abstract] OR "robot therapy sessions"[Title/Abstract] OR "social robot"[Title/Abstract] OR "socially assistive robot"[Title/Abstract] OR "robotic pet"[Title/Abstract] OR "robotic therapy"[Title/Abstract] OR "robot assisted therapy"[Title/Abstract] OR "robot assisted therapies"[Title/Abstract])) | 127,892 |
| 9 | "robotics/therapeutic use"[MeSH Terms] OR "robotics/therapy"[MeSH Terms] OR "robot therapy"[Title/Abstract] OR "robot therapy sessions"[Title/Abstract] OR "social robot"[Title/Abstract] OR "socially assistive robot"[Title/Abstract] OR "robotic pet"[Title/Abstract] OR "robotic therapy"[Title/Abstract] OR "robot assisted therapy"[Title/Abstract] OR "robot assisted therapies"[Title/Abstract] | 3,229 |
| 8 | "robot therapy"[Title/Abstract] OR "robot therapy sessions"[Title/Abstract] OR "social robot"[Title/Abstract] OR "socially assistive robot"[Title/Abstract] OR "robotic pet"[Title/Abstract] OR "robotic therapy"[Title/Abstract] OR "robot assisted therapy"[Title/Abstract] OR "robot assisted therapies"[Title/Abstract] | 775 |
| 7 | "robotics/therapeutic use"[MeSH Terms] OR "robotics/therapy"[MeSH Terms] | 2,454 |
| 6 | "Animal Assisted Therapy"[MeSH Terms] OR "Pets"[MeSH Terms] OR ("animal assisted therapies"[Title/Abstract] OR "animal facilitated therapy"[Title/Abstract] OR (("animals"[MeSH Terms:noexp] OR "Animal"[All Fields]) AND "facilitated therapies"[Title/Abstract]) OR "Animal Assisted Therapy"[Title/Abstract] OR "pet therapy"[Title/Abstract] OR "pet therapies"[Title/Abstract] OR "pet facilitated therapy"[Title/Abstract] OR ("Pet"[All Fields] AND "facilitated therapies"[Title/Abstract]) OR "pet assisted therapy"[Title/Abstract] OR ("Pet-Assisted"[All Fields] AND "Therapies"[Title/Abstract]) OR "pet assisted therapy"[Title/Abstract] OR ("Pet"[All Fields] AND "assisted therapies"[Title/Abstract]) OR "Pet"[Title/Abstract] OR "companion animal"[Title/Abstract] OR "companion cat"[Title/Abstract] OR "companion dog"[Title/Abstract]) | 124,692 |
| 5 | "animal assisted therapies"[Title/Abstract] OR "animal facilitated therapy"[Title/Abstract] OR (("animals"[MeSH Terms:noexp] OR "Animal"[All Fields]) AND "facilitated therapies"[Title/Abstract]) OR "animal assisted therapy"[Title/Abstract] OR "pet therapy"[Title/Abstract] OR "pet therapies"[Title/Abstract] OR "pet facilitated therapy"[Title/Abstract] OR ("Pet"[All Fields] AND "facilitated therapies"[Title/Abstract]) OR "pet assisted therapy"[Title/Abstract] OR ("Pet-Assisted"[All Fields] AND "Therapies"[Title/Abstract]) OR "pet assisted therapy"[Title/Abstract] OR ("Pet"[All Fields] AND "assisted therapies"[Title/Abstract]) OR "Pet"[Title/Abstract] OR "companion animal"[Title/Abstract] OR "companion cat"[Title/Abstract] OR "companion dog"[Title/Abstract] | 122,584 |
| 4 | "Animal Assisted Therapy"[MeSH Terms] OR "Pets"[MeSH Terms] | 3,916 |
| 3 | "Dementia"[MeSH Terms] OR "Alzheimer Disease"[MeSH Terms] OR "Dementia"[Title/Abstract] OR "Amentia"[Title/Abstract] OR "Alzheimer Disease"[Title/Abstract] OR "alzheimer s disease"[Title/Abstract] OR "alzheimer sclerosis"[Title/Abstract] OR "alzheimer syndrome"[Title/Abstract] | 294,260 |
| 2 | "Dementia"[Title/Abstract] OR "Amentia"[Title/Abstract] OR "alzheimer disease"[Title/Abstract] OR "alzheimer’s disease"[Title/Abstract] OR "alzheimer sclerosis"[Title/Abstract] OR "alzheimer syndrome"[Title/Abstract] | 249,116 |
| 1 | "Dementia"[MeSH Terms] OR "Alzheimer Disease"[MeSH Terms] | 195,290 |

*Embase*

| ID | Query | Results |
| --- | --- | --- |
| #11 | #3 AND #7 AND #10 | 98 |
| #10 | #8 OR #9 | 2092587 |
| #9 | 'randomized controlled trial'/exp OR 'randomized controlled trial (topic)'/exp OR 'randomization'/exp | 1033561 |
| #8 | random* | 2091632 |
| #7 | #4 OR #5 OR #6 | 34626 |
| #6 | 'animal assisted therapy'/exp OR 'pet animal'/exp OR 'robotics'/exp/mj | 32610 |
| #5 | 'robot therapy':ti,ab,kw OR 'robot therapy sessions':ti,ab,kw OR 'social robot':ti,ab,kw OR 'socially assistive robot':ti,ab,kw OR 'robotic pet':ti,ab,kw OR 'robotic therapy':ti,ab,kw OR 'robot assisted therapy':ti,ab,kw OR 'robot assisted therapies':ti,ab,kw | 921 |
| #4 | 'animal assisted therapies':ti,ab,kw OR 'animal facilitated therapies':ti,ab,kw OR 'animal assisted therapy':ti,ab,kw OR 'pet therapy':ti,ab,kw OR 'pet therapies':ti,ab,kw OR 'pet facilitated therapy':ti,ab,kw OR 'pet facilitated therapies':ti,ab,kw OR 'pet-assisted therapy':ti,ab,kw OR 'pet-assisted therapies':ti,ab,kw OR 'pet assisted therapy':ti,ab,kw OR 'pet assisted therapies':ti,ab,kw OR 'companion animal':ti,ab,kw OR 'pet animal':ti,ab,kw OR 'companion cat':ti,ab,kw OR 'companion dog':ti,ab,kw | 2535 |
| #3 | #1 OR #2 | 453826 |
| #2 | 'dementia'/exp OR 'alzheimer disease'/exp | 418388 |
| #1 | dementia:ti,ab,kw OR amentia:ti,ab,kw OR 'alzheimer disease':ti,ab,kw OR 'alzheimer sclerosis':ti,ab,kw OR 'alzheimer syndrome':ti,ab,kw | 211329 |

*Cochrane library*

| ID | Search | Hits |
| --- | --- | --- |
| #1 | (Dementia):ti,ab,kw OR (Amentia):ti,ab,kw OR ("Alzheimer disease"):ti,ab,kw OR ("Alzheimer's disease"):ti,ab,kw OR (''Alzheimer Sclerosis''):ti,ab,kw | 21961 |
| #2 | (''Alzheimer Syndrome''):ti,ab,kw | 461 |
| #3 | #1 or #2 | 21982 |
| #4 | MeSH descriptor: [Dementia] this term only | 2805 |
| #5 | MeSH descriptor: [Alzheimer Disease] this term only | 3795 |
| #6 | #3 or #4 or #5 | 21982 |
| #7 | (''Animal Assisted Therapies''):ti,ab,kw OR (''Animal Facilitated Therapy''):ti,ab,kw OR (''Animal Facilitated Therapies''):ti,ab,kw OR ("animal assisted therapy"):ti,ab,kw OR (''Pet Therapy''):ti,ab,kw | 3934 |
| #8 | (''Pet Therapies''):ti,ab,kw OR (''Pet Facilitated Therapy''):ti,ab,kw OR (''Pet Facilitated Therapies''):ti,ab,kw OR (''Pet-Assisted Therapy''):ti,ab,kw OR (''Pet-Assisted Therapies''):ti,ab,kw | 326 |
| #9 | (''Pet Assisted Therapy''):ti,ab,kw OR (''Pet Assisted Therapies''):ti,ab,kw OR (''Companion Animal''):ti,ab,kw OR (''Companion Cat''):ti,ab,kw | 328 |
| #10 | (''Companion Dog''):ti,ab,kw | 21 |
| #11 | #7 or #8 or #9 or #10 | 4016 |
| #12 | MeSH descriptor: [Animal Assisted Therapy] this term only | 47 |
| #13 | MeSH descriptor: [Pets] this term only | 15 |
| #14 | #11 or #12 or #13 | 4025 |
| #15 | (robotic*):ti,ab,kw OR (robot therapy):ti,ab,kw OR (social robot):ti,ab,kw OR (robot-assisted):ti,ab,kw | 5879 |
| #16 | MeSH descriptor: [Robotics] explode all trees | 1116 |
| #17 | #15 or #16 | 5879 |
| #18 | #11 or #17 | 9858 |
| #19 | (random*):ti,ab,kw | 1167879 |
| #20 | MeSH descriptor: [Randomized Controlled Trial] this term only | 118 |
| #21 | MeSH descriptor: [Randomized Controlled Trials as Topic] this term only | 12817 |
| #22 | MeSH descriptor: [Random Allocation] this term only | 20678 |
| #23 | #19 or #20 or #21 or #22 | 1167880 |
| #24 | #6 and #18 and #23 in Trials | 297 |

*Web of Science*

| ID | Query | Results |
| --- | --- | --- |
| #1 | 1: (((((AB=(Dementia)) OR AB=(Amentia)) OR AB=(Alzheimer Disease)) OR AB=(Alzheimer's Disease)) OR AB=(Alzheimer Sclerosis)) OR AB=(Alzheimer Syndrome) | 294760 |
| #2 | 2: ((((((((((((((((((((((AB=(Animal Assisted Therapies)) OR AB=(Animal Assisted Therapies)) OR AB=(Animal Assisted Therapies)) OR AB=(Animal Assisted Therapy)) OR AB=(Pet Therapy)) OR AB=(Pet Therapies)) OR AB=(Pet Facilitated Therapy)) OR AB=(Pet Facilitated Therapies)) OR AB=(Pet-Assisted Therapy)) OR AB=(Pet-Assisted Therapies)) OR AB=(Pet Assisted Therapy)) OR AB=(Pet Assisted Therapies)) OR AB=(Companion Animal)) OR AB=(Companion Cat)) OR AB=(Companion Dog)) OR AB=(Robot therapy)) OR AB=(robot therapy sessions)) OR AB=(social robot)) OR AB=(socially assistive robot)) OR AB=(robotic pet)) OR AB=(robotic therapy)) OR AB=(robot assisted therapy)) OR AB=(robot assisted therapies) | 45192 |
| #3 | 3: AB=(random*) | 2363954 |
| #4 | 4: #3 AND #2 AND #1 | 67 |

*SCOPUS*

| ID | Query | Results |
| --- | --- | --- |
| #1 | (TITLE-ABS-KEY ( dementia ) OR TITLE-ABS-KEY ( amentia ) OR TITLE-ABS-KEY ( alzheimer AND disease ) OR TITLE-ABS-KEY ( alzheimer's AND disease ) OR TITLE-ABS-KEY ( alzheimer AND sclerosis ) OR TITLE-ABS-KEY ( alzheimer AND syndrome ) ) | 392,374 |
| #2 | (TITLE-ABS-KEY ( animal AND assisted AND therapies ) OR TITLE-ABS-KEY ( animal AND facilitated AND therapy ) OR TITLE-ABS-KEY ( animal AND facilitated AND therapies ) OR TITLE-ABS-KEY ( animal AND assisted AND therapy ) OR TITLE-ABS-KEY ( pet AND therapy ) OR TITLE-ABS-KEY ( pet AND therapies ) OR TITLE-ABS-KEY ( pet AND facilitated AND therapy ) OR TITLE-ABS-KEY ( pet AND facilitated AND therapies ) OR TITLE-ABS-KEY ( pet-assisted AND therapy ) OR TITLE-ABS-KEY ( pet-assisted AND therapies ) OR TITLE-ABS-KEY ( pet AND assisted AND therapy ) OR TITLE-ABS-KEY ( pet AND assisted AND therapies ) OR TITLE-ABS-KEY ( companion AND animal ) OR TITLE-ABS-KEY ( companion AND cat ) OR TITLE-ABS-KEY ( companion AND dog ) OR TITLE-ABS-KEY ( robot AND therapy ) OR TITLE-ABS-KEY ( robot AND therapy AND sessions ) OR TITLE-ABS-KEY ( social AND robot ) OR TITLE-ABS-KEY ( socially AND assistive AND robot ) OR TITLE-ABS-KEY ( robotic AND pet ) OR TITLE-ABS-KEY ( robotic AND therapy ) OR TITLE-ABS-KEY ( robot AND assisted AND therapy ) OR TITLE-ABS-KEY ( robot AND assisted AND therapies ) ) | 95,535 |
| #3 | ALL ( random* ) | 8,021,302 |
| #4 | ( ( TITLE-ABS-KEY ( dementia ) OR TITLE-ABS-KEY ( amentia ) OR TITLE-ABS-KEY ( alzheimer AND disease ) OR TITLE-ABS-KEY ( alzheimer's AND disease ) OR TITLE-ABS-KEY ( alzheimer AND sclerosis ) OR TITLE-ABS-KEY ( alzheimer AND syndrome ) ) ) AND ( ( TITLE-ABS-KEY ( animal AND assisted AND therapies ) OR TITLE-ABS-KEY ( animal AND facilitated AND therapy ) OR TITLE-ABS-KEY ( animal AND facilitated AND therapies ) OR TITLE-ABS-KEY ( animal AND assisted AND therapy ) OR TITLE-ABS-KEY ( pet AND therapy ) OR TITLE-ABS-KEY ( pet AND therapies ) OR TITLE-ABS-KEY ( pet AND facilitated AND therapy ) OR TITLE-ABS-KEY ( pet AND facilitated AND therapies ) OR TITLE-ABS-KEY ( pet-assisted AND therapy ) OR TITLE-ABS-KEY ( pet-assisted AND therapies ) OR TITLE-ABS-KEY ( pet AND assisted AND therapy ) OR TITLE-ABS-KEY ( pet AND assisted AND therapies ) OR TITLE-ABS-KEY ( companion AND animal ) OR TITLE-ABS-KEY ( companion AND cat ) OR TITLE-ABS-KEY ( companion AND dog ) OR TITLE-ABS-KEY ( robot AND therapy ) OR TITLE-ABS-KEY ( robot AND therapy AND sessions ) OR TITLE-ABS-KEY ( social AND robot ) OR TITLE-ABS-KEY ( socially AND assistive AND robot ) OR TITLE-ABS-KEY ( robotic AND pet ) OR TITLE-ABS-KEY ( robotic AND therapy ) OR TITLE-ABS-KEY ( robot AND assisted AND therapy ) OR TITLE-ABS-KEY ( robot AND assisted AND therapies ) ) ) AND ( ALL ( random* ) ) | 624 |
| #5 | ( ( TITLE-ABS-KEY ( dementia ) OR TITLE-ABS-KEY ( amentia ) OR TITLE-ABS-KEY ( alzheimer AND disease ) OR TITLE-ABS-KEY ( alzheimer's AND disease ) OR TITLE-ABS-KEY ( alzheimer AND sclerosis ) OR TITLE-ABS-KEY ( alzheimer AND syndrome ) ) ) AND ( ( TITLE-ABS-KEY ( animal AND assisted AND therapies ) OR TITLE-ABS-KEY ( animal AND facilitated AND therapy ) OR TITLE-ABS-KEY ( animal AND facilitated AND therapies ) OR TITLE-ABS-KEY ( animal AND assisted AND therapy ) OR TITLE-ABS-KEY ( pet AND therapy ) OR TITLE-ABS-KEY ( pet AND therapies ) OR TITLE-ABS-KEY ( pet AND facilitated AND therapy ) OR TITLE-ABS-KEY ( pet AND facilitated AND therapies ) OR TITLE-ABS-KEY ( pet-assisted AND therapy ) OR TITLE-ABS-KEY ( pet-assisted AND therapies ) OR TITLE-ABS-KEY ( pet AND assisted AND therapy ) OR TITLE-ABS-KEY ( pet AND assisted AND therapies ) OR TITLE-ABS-KEY ( companion AND animal ) OR TITLE-ABS-KEY ( companion AND cat ) OR TITLE-ABS-KEY ( companion AND dog ) OR TITLE-ABS-KEY ( robot AND therapy ) OR TITLE-ABS-KEY ( robot AND therapy AND sessions ) OR TITLE-ABS-KEY ( social AND robot ) OR TITLE-ABS-KEY ( socially AND assistive AND robot ) OR TITLE-ABS-KEY ( robotic AND pet ) OR TITLE-ABS-KEY ( robotic AND therapy ) OR TITLE-ABS-KEY ( robot AND assisted AND therapy ) OR TITLE-ABS-KEY ( robot AND assisted AND therapies ) ) ) AND ( ALL ( random* ) ) | 322 |
