# Supplementary material for: Network meta-analysis of comparative efficacy of animal-assisted therapy vs. pet-robot therapy in the management of dementia
Source: Front Aging Neurosci. 2023 May 31;15:1095996. doi: 10.3389/fnagi.2023.1095996 (PMC10264590; doi:10.3389/fnagi.2023.1095996)
Supplement: Supplementary file 2 [file Table_2.DOCX]

**Supplementary Table 2.** The detailed information of AAT and PRT of the included studies.

| **Study** | **Detailed interventions** | **Frequency** |
| --- | --- | --- |
| Travers et.al., 2013 | Interaction with the dog through play, petting and/or feeding it, and concluded by reading a short story to the group. | 40~50 min/session, 3 days/week for 11 weeks |
| Bono et.al., 2015 | Contact with dog for cognitive stimulation, communication, motor activity, and wellbeing and entertainment. | Twice weekly one-hour sessions for 8 months |
| Friedmann et.al., 2015 | No specific mention on formal training of the facilitators on the use of therapy animal. | 60~90 min/session, twice weekly for 12 weeks |
| Olsen et.al., 2016a | The participants were randomly seated in a half-circle, and the dog handler moved around the group so that each participant was able to greet the dog and feed it treats. Next, the handler organized different activities such as petting the dog, brushing the dog, feeding the dog a treat, or throwing a toy for the dog to fetch. | 30 min/session, 2 times/week for 12 weeks |
| Olsen et.al., 2016b | The participants were randomly seated in a half-circle. Each session started with a greeting round, when each participant had the opportunity to pet the dog and feed it treats. Thereafter, the handler started the different activities, which included any of the following: petting the dog, feeding the dog a treat and throwing a toy for the dog to fetch. | 30 min/session, 2 times/week for 12 weeks |
| Briones et.al., 2021 | Receive activities focused on cognitive and motor functions, communication, recreational pursuits, and well-being. | Weekly 50 min sessions for 9 months |
| Pope et.al., 2016 | Interactions through touching, petting, brushing, holding, talking to and playing with the dogs. | 10 min visit, twice weekly for 2 weeks |
| Vegue Parra et.al., 2021 | Affective area: caressing and brushing, positive communication towards the dog and the participants; Behavioral area: varied activities with dog; Functional area: coordination and fine motor skills with the dog; Cognitive area: work on reminiscence and memory stimulation related to animals from their past. Language and thought stimulation through word formation games, sayings or categorization with the dog as the central element of the activity. | 8 months, with weekly sessions of 45 min |
| Quintavalla et.al., 2021 | Introducing the dog; Nose work with objects; Solitaire dog games; Image search and recognition; home-made and industrial feeding; Care and massage; Doggy brain train; Standard mobility path; home-made mobility path; Recalling favorite activity mostly appreciated by the single group. | A total of 24 sessions over a span of 12 weeks |
| Jøranson et al., 2015 | Petting, talking to and about, smiling to, and singing to the robotic animal. | 30 min/session, 2 times/week for 12 weeks |
| Jøranson et al., 2016 | Petting, talking to and about, smiling to, and singing to the robotic animal. | 30 min/session, 2 times/week for 12 weeks |
| Liang et.al., 2017 | Interactions with PARO, such as stroking PARO’s flippers. | 30 min/session, 2-3 times/weeks for 6 weeks |
| Moyle et.al., 2013 | Involved activities around the concepts of discovery, engaging an emotional response, social interaction in the group through discussion about PARO, and touching PARO. | 45 min/session, 3afternoons/week for 5 weeks |
| Moyle et.al., 2017 | Participants interacted with PARO as they liked. | 15 min/session, 3 times/week for 10 weeks |
| Moyle et.al., 2019 | Interaction with lifelike baby doll at times outside of routine meal and rest periods. | 30 min/session, 3 days/week for 3 weeks |
| Petersen et al., 2017 | Interaction activity of 6 people one group to PARO. | 20 min/session, 3 days/week for 3 months |
| Pu et al., 2020 | Interaction with individual non-facilitated PARO in bedrooms. | 30 minute/session, 5 days/week for 6 weeks |
| Robinson et.al., 2013 | During sessions with the robot, discussion groups were held and all residents had a chance to interact with the robot. | 1 hour/session, 2 afternoons/week for 12 weeks |
| Valenti Soler et.al., 2015 | interaction with the dog through play, petting and/or feeding it, and concluded by reading a short story to the group. | 30~40 min/session, 2 times/week for 3 months |
|  | Interactions with PAROs to perform several therapeutic activities, including identifying numbers, words, and colors using flash cards. | 30~40 min/session, 2 times/week for 3 months |

AAT, animal-assisted therapy; PRT, pet-robot therapy.
