# Supplementary material for: Network meta-analysis of comparative efficacy of animal-assisted therapy vs. pet-robot therapy in the management of dementia
Source: Front Aging Neurosci. 2023 May 31;15:1095996. doi: 10.3389/fnagi.2023.1095996 (PMC10264590; doi:10.3389/fnagi.2023.1095996)
Supplement: Supplementary file 3 [file Table_3.DOCX]

**Supplementary Table 3.** Outcomes and measurements of the included studies.

| Study | Group | Agitation | Cognitive function | Depression | QoL | Measurements |
| --- | --- | --- | --- | --- | --- | --- |
| Travers et.al., 2013 | Dog | n.a. | n.a. | 12.5±3.7 | 35.42±5.78 | Modified Mini-Mental State Exam for depression, and Quality of life in Alzheimer's Disease for QoL. |
|  | Control | n.a. | n.a. | 14.6±3.9 | 37.47±2.62 |  |
| Bono et.al., 2015 | Dog | n.a. | 33±12.89 | 8.1±7.97 | n.a. | ADAS for cognitive function, and Cornell scale for depression in dementia for depression. |
|  | Control | n.a. | 35±13.79 | 4.4±4.85 | n.a. |  |
| Friedmann et.al., 2015 | Dog | 15.53±2.96 | n.a. | 5.21±3.36 | n.a. | Cornell scale for depression in dementia for depression, and Cohen-Mansfield agitation inventory for agitation. |
|  | Control | 20.00±7.17 | n.a. | 8.67±6.41 | n.a. |  |
| Olsen et.al., 2016a | Dog | n.a. | n.a. | n.a. | 16.52±6.9 | Quality of life in late-stage dementia for QoL. |
|  | Control | n.a. | n.a. | n.a. | 16.28±4.28 |  |
| Olsen et.al., 2016b | Dog | 23.75±7.13 | n.a. | 7.86±4.42 | 25.31±10.26 | Cornell scale for depression in dementia for depression, Brief agitation rating scale for agitation, and Quality of life in late-stage dementia for QoL. |
|  | Control | 24.65±13.95 | n.a. | 8.28±5.62 | 24.8±5.79 |  |
| Briones et.al., 2021 | Dog | n.a. | n.a. | n.a. | 30.74±3.6 | Quality of life in Alzheimer's Disease Scale for QoL. |
|  | Control | n.a. | n.a. | n.a. | 28.62±3.78 |  |
| Pope et.al., 2016 | Dog | 34.00±12.8 | n.a. | n.a. | n.a. | Cohen-Mansfield Agitation Inventory for agitation. |
|  | Control | 36.60±13.40 | n.a. | n.a. | n.a. |  |
| Vegue Parra et.al., 2021 | Dog | n.a. | 18.48±1.39 | 5.38±5.97 | n.a. | Mini-Mental State Examination for cognitive function, and Cornell scale for Depression in Dementia for depression. |
|  | Control | n.a. | 19.38±2.02 | 8.88±7.09 | n.a. |  |
| Quintavalla et.al., 2021 | Dog | n.a. | 13.18±7.86 | n.a. | n.a. | Alzheimer’s Disease Assessment Scale for depression |
|  | Control | n.a. | 12.23±7.23 | n.a. | n.a. |  |
| Jøranson et al., 2015 | PARO | 18.2±7.0 | n.a. | 7.2±6.4 | n.a. | Brief Agitation Rating Scale for agitation, and Cornell scale for depression in dementia for depression. |
|  | Control | 24.0±13.2 | n.a. | 9.3±6.6 | n.a. |  |
| Jøranson et al., 2016 | PARO | n.a. | 26.65±10.17 | n.a. | 23.76±7.22 | Cohen-Mansfield Agitation Inventory-Short Form for agitation, Brief Agitation Rating Scale for agitation, and Cornell scale for depression in dementia for depression. |
|  | Control | n.a. | 23.76±7.23 | n.a. | 26.48±10.05 |  |
| Liang et.al., 2017 | PARO | 26.410.4 | 37.9±19.2 | 7.77±6.72 | n.a. | Addenbrooke’s Cognitive Examination for cognitive function, Cornell Scale for Depression in Dementia for depression, Cohen-Mansfield Agitation Inventory Short Form for agitation. |
|  | Control | 24.77.16 | 37.6±20.9 | 5.18±3.06 | n.a. |  |
| Moyle et.al., 2013 | PARO | 46.2±12.2 | n.a. | 4.7±2.9 | 37.2±8.2 | Geriatric Depression Scale for depression, Revised Algase Wandering Scale-Nursing  Home version for agitation, and Quality of Life in Alzheimer's Disease Scale for QoL. |
|  | Control | 46.8±13.0 | n.a. | 4.3±3.5 | 26.4±16.8 |  |
| Moyle et.al., 2017 | PARO | 26.34±6.15 | n.a. | n.a. | n.a. | Cohen-Mansfield Agitation Inventory-Short Form for agitation. |
|  | Control | 32.32±7.56 | n.a. | n.a. | n.a. |  |
| Moyle et.al., 2019 | Lifelike toll | 16.28±3.53 | n.a. | n.a. | n.a. | Cohen-Mansfield Agitation Inventory-Short Form for agitation. |
|  | Control | 16.20±2.83 | n.a. | n.a. | n.a. |  |
| Petersen et al., 2017 | PARO | n.a. | n.a. | 15.31±6.7 | n.a. | Cornell Scale for Depression in Dementia for depression. |
|  | Control | n.a. | n.a. | 10.18±4.8 | n.a. |  |
| Pu et al., 2020 | PARO | 26.95±11.84 | n.a. | 9.57±8.38 | n.a. | Cornell Scale for Depression in Dementia for depression, and Cohen-Mansfield Agitation Inventory-Short for agitation. |
|  | Control | 31.82±15.15 | n.a. | 14.32±9.62 | n.a. |  |
| Robinson et.al., 2013 | PARO | n.a. | n.a. | 4.15±2.34 | 32.73±8.24 | Geriatric Depression Scale for depression, and Quality of Life for Alzheimer’s Disease for QoL. |
|  | Control | n.a. | n.a. | 4.00±2.62 | 31.19±6.26 |  |
| Valenti Soler et.al., 2015 | Dog | n.a. | n.a. | n.a. | 24.33±6.68 | Quality of life in late-stage dementia for QoL. |
|  | PARO | n.a. | n.a. | n.a. | 26.75±8.16 |  |
|  | Control | n.a. | n.a. | n.a. | 24.72±6.68 |  |

QoL, quality of life; n.a, not available.
