# Supplementary material for: Network meta-analysis of comparative efficacy of animal-assisted therapy vs. pet-robot therapy in the management of dementia
Source: Front Aging Neurosci. 2023 May 31;15:1095996. doi: 10.3389/fnagi.2023.1095996 (PMC10264590; doi:10.3389/fnagi.2023.1095996)
Supplement: Supplementary file 4 [file Table_4.docx]

**Supplementary Table 4.** Transitivity assessment between the included studies.

| Variables | Comparisons | Mean*/χ^2^* | *SE/df* | *P* |
| --- | --- | --- | --- | --- |
| Origin | AAT *vs.* Control | 2.387 | 2 | 0.303 |
|  | PRT *vs.* Control | 1.421 | 2 | 0.491 |
|  | AAT *vs.* PRT | 5.505 | 2 | 0.064 |
| Publication year | AAT *vs.* Control | 0.268 | 1.077 | 0.805 |
|  | PRT *vs.* Control | -0.432 | 0.992 | 0.667 |
|  | AAT *vs.* PRT | 0.700 | 1.186 | 0.562 |
| Sample size, n | AAT *vs.* Control | 6.095 | 16.839 | 0.720 |
|  | PRT *vs.* Control | -0.505 | 15.611 | 0.974 |
|  | AAT *vs.* PRT | 6.600 | 18.790 | 0.729 |
| Mean age, years | AAT *vs.* Control | -3.135 | 3.490 | 0.376 |
|  | PRT *vs.* Control | 4.261 | 3.276 | 0.204 |
|  | AAT *vs.* PRT | -7.400 | 3.900 | 0.074 |
| Gender ratio, % | AAT *vs.* Control | -0.030 | 0.063 | 0.642 |
|  | PRT *vs.* Control | 0.105 | 0.067 | 0.136 |
|  | AAT *vs.* PRT | -0.135 | 0.066 | 0.065 |
| Dementia stage, MMSE* | AAT *vs.* Control | -0.061 | 0.069 | 0.387 |
|  | PRT *vs.* Control | 0.177 | 0.080 | 0.052 |
|  | AAT *vs.* PRT | -0.238 | 0.055 | 0.061 |

AAT, animal-assisted therapy; PRT, pet robot therapy; MMSE, Mini Mental State Examination; SE, standard error; NA, not available; *df*, degree of freedom. *We calculated standardized score for dementia stage by dividing the actual score by the overall score for each scale.
