# Supplementary figures and images for: Network meta-analysis of comparative efficacy of animal-assisted therapy vs. pet-robot therapy in the management of dementia
Source: Front Aging Neurosci. 2023 May 31;15:1095996. doi: 10.3389/fnagi.2023.1095996 (PMC10264590; doi:10.3389/fnagi.2023.1095996)

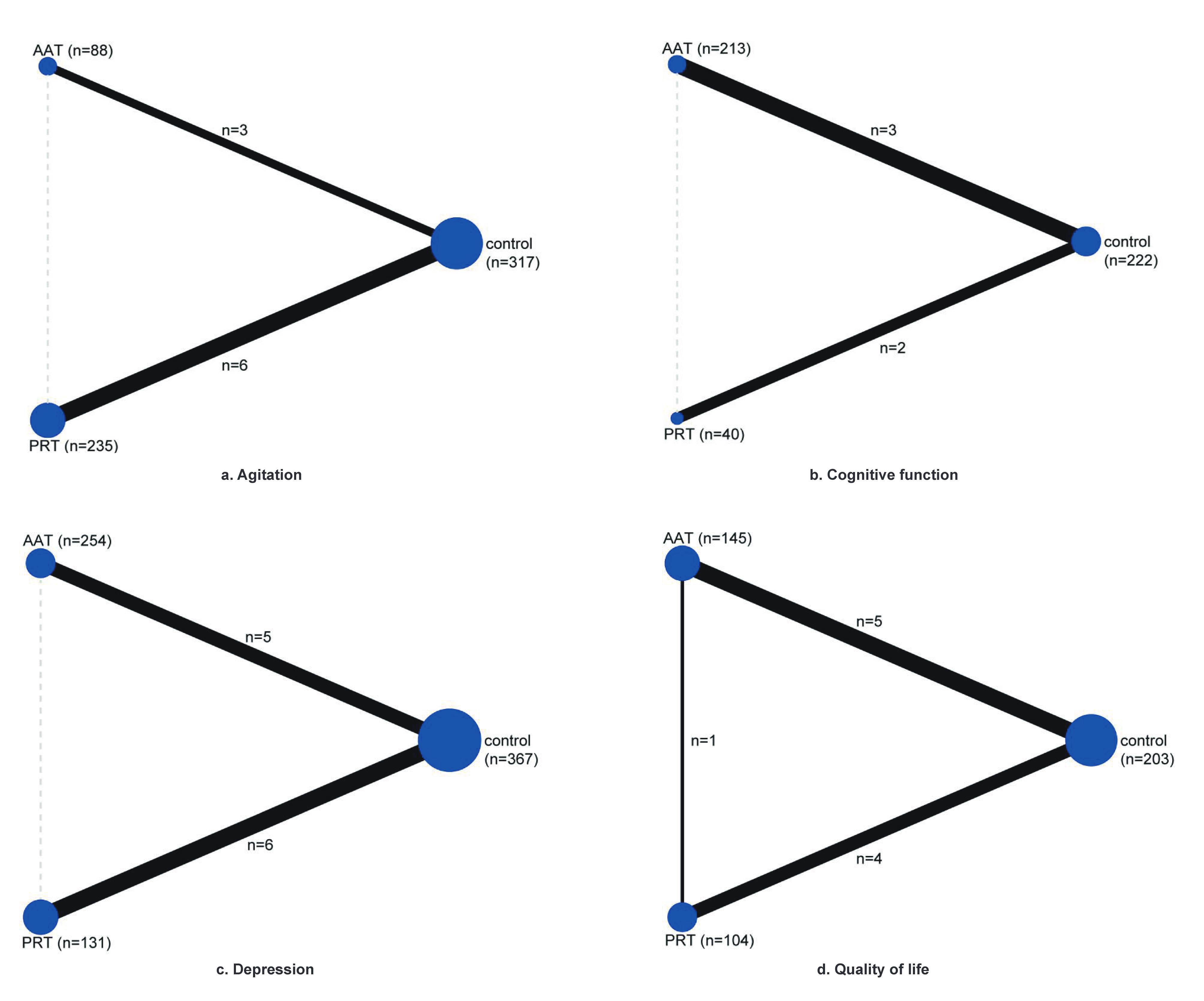

Supplement: Supplementary file 5 [file Image_1.JPEG]

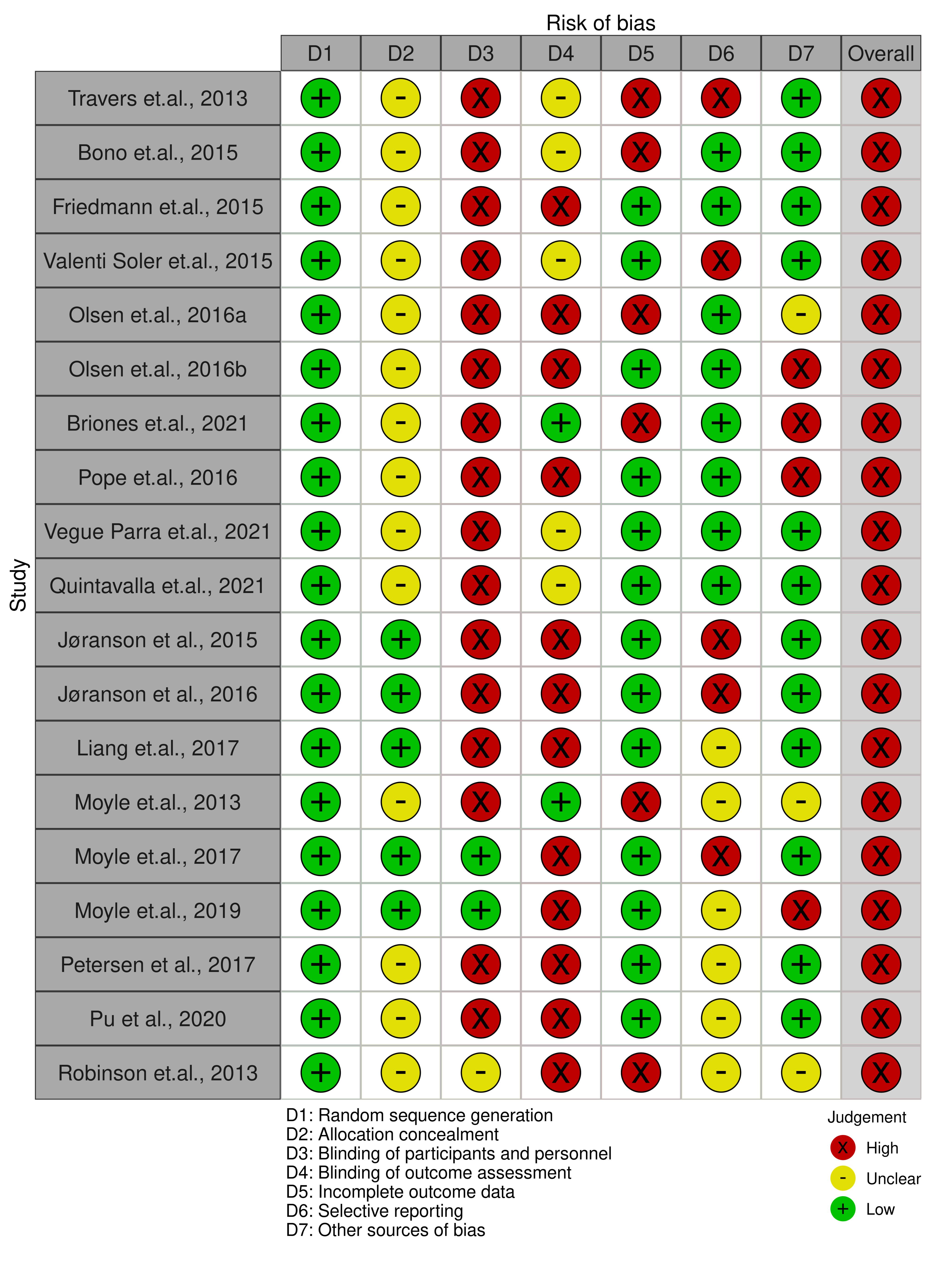

Supplement: Supplementary file 6 [file Image_2.JPEG]

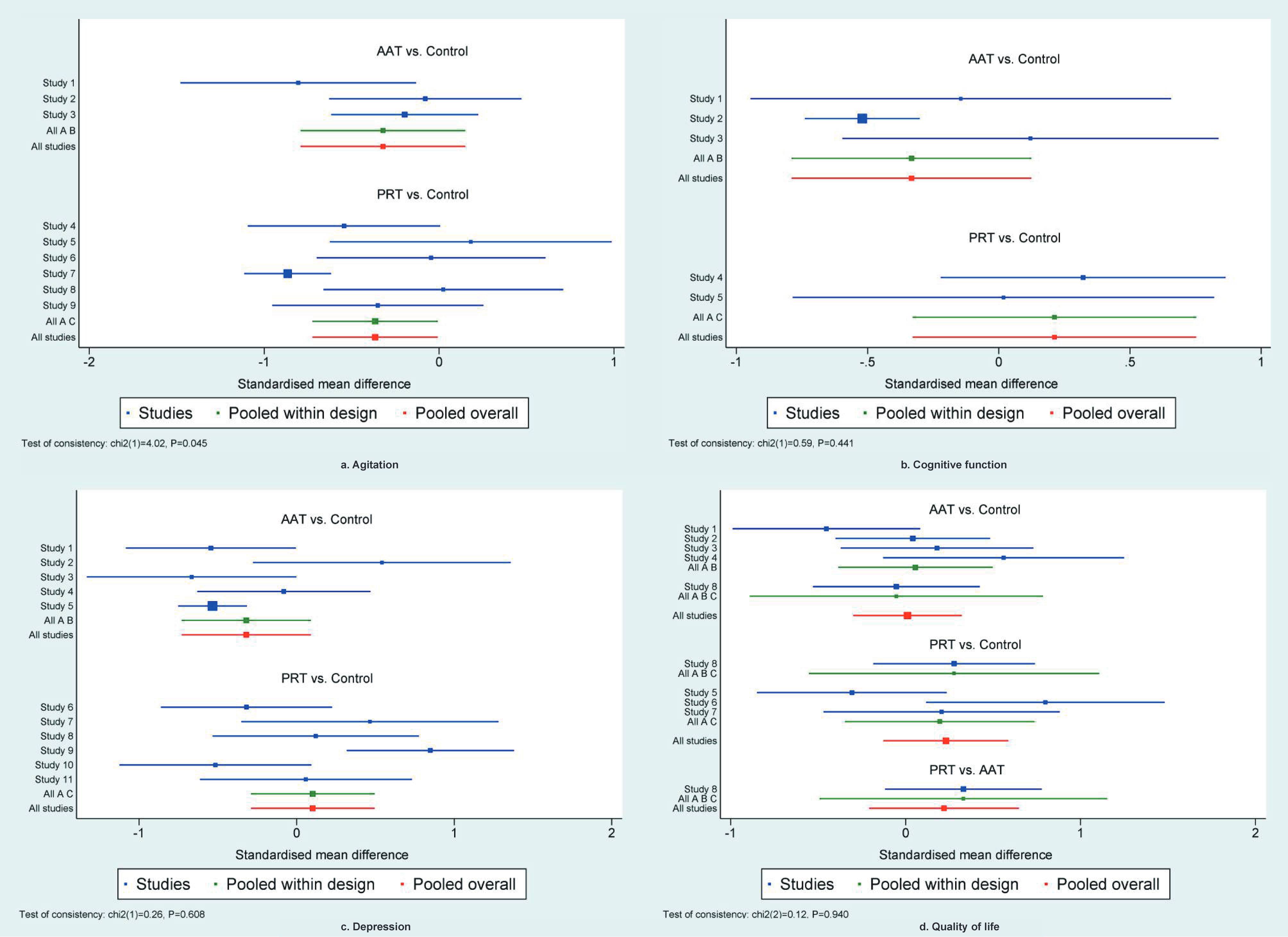

Supplement: Supplementary file 7 [file Image_3.JPEG]

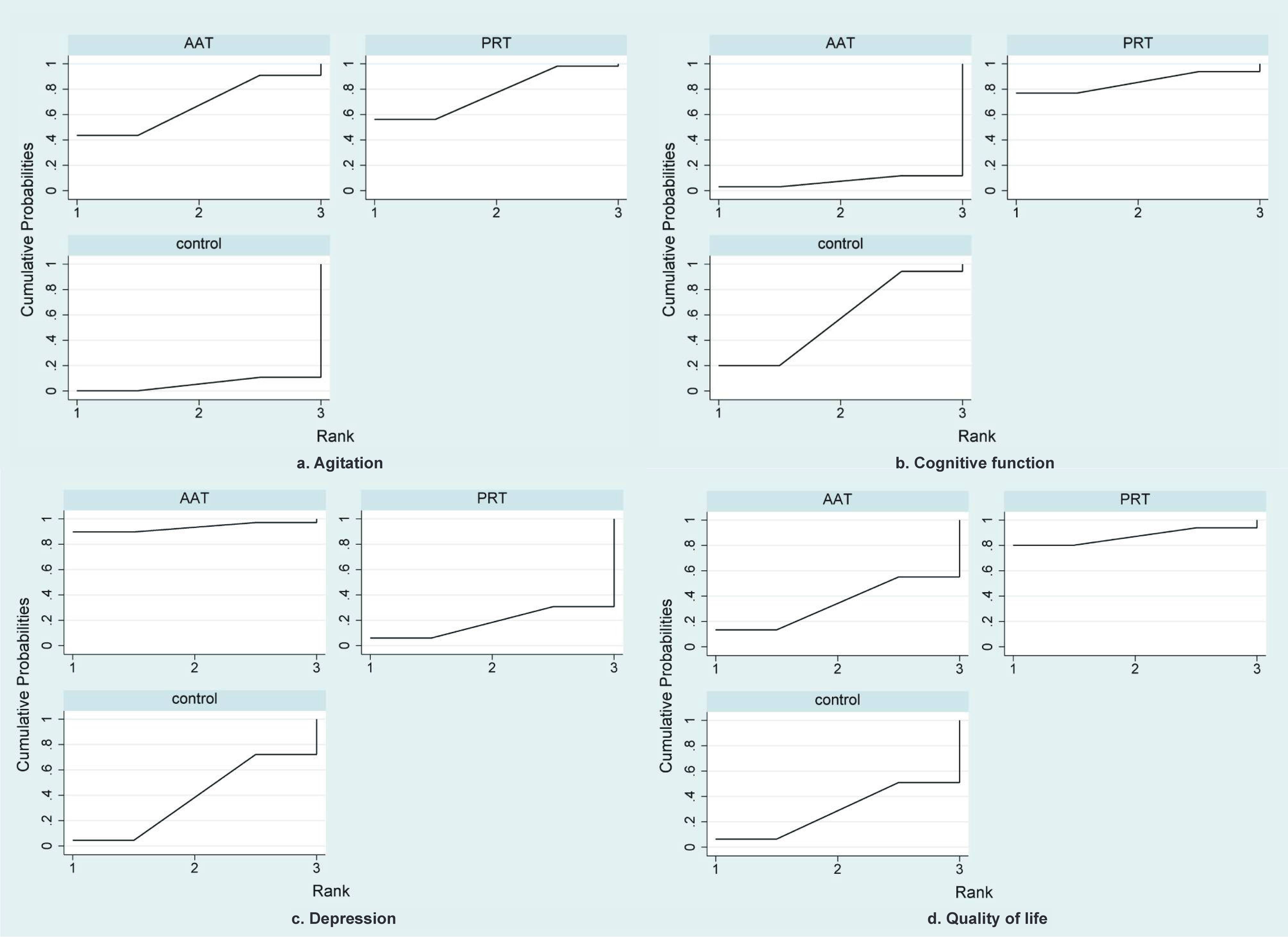

Supplement: Supplementary file 8 [file Image_4.JPEG]

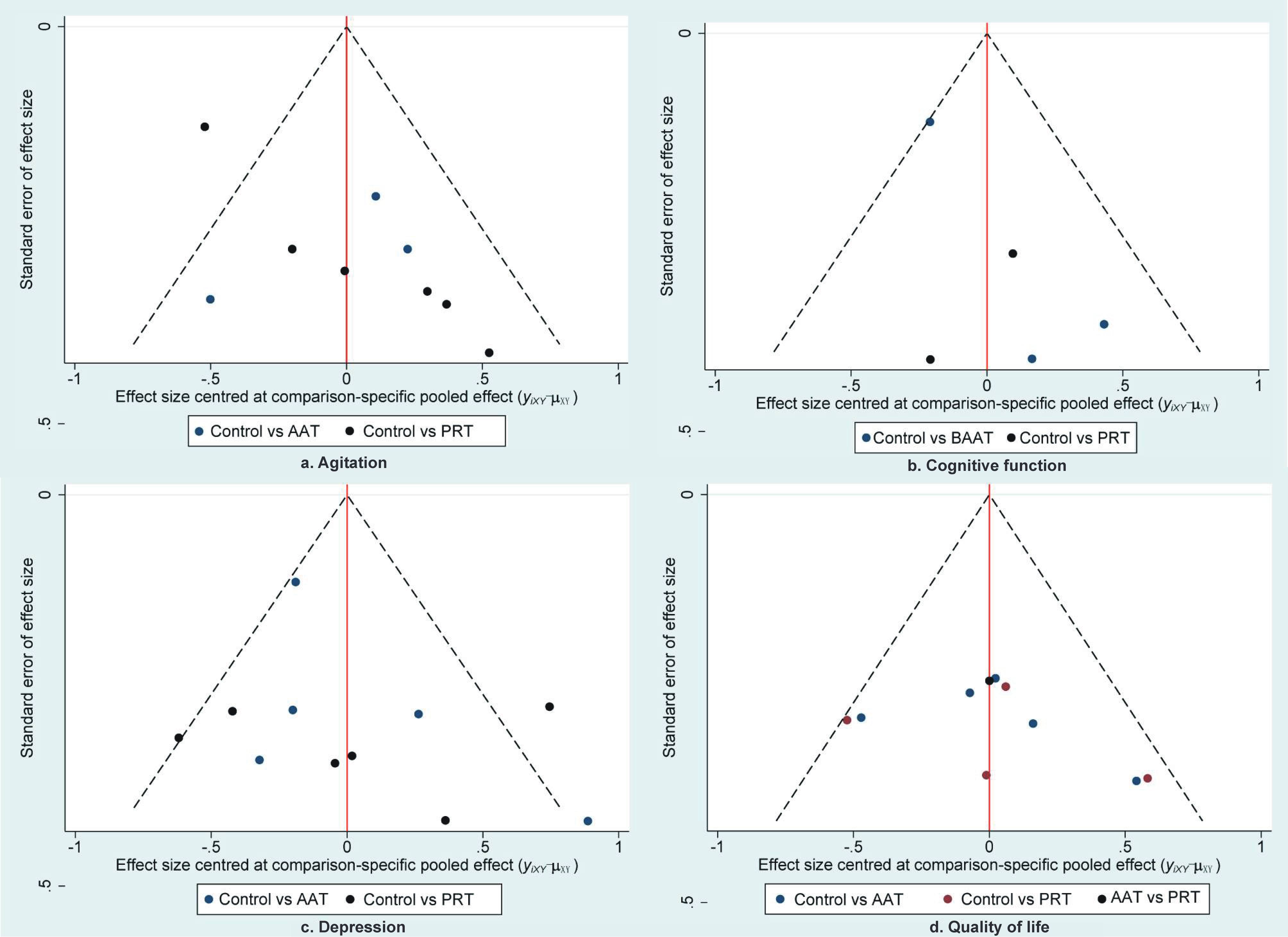

Supplement: Supplementary file 9 [file Image_5.JPEG]
